# Abnormal lipid metabolism in epidermal Langerhans cells mediates psoriasis-like dermatitis

**DOI:** 10.1172/jci.insight.150223

**Published:** 2022-07-08

**Authors:** Xilin Zhang, Xiaorui Li, Yuanyuan Wang, Youdong Chen, Yijun Hu, Chunyuan Guo, Zengyang Yu, Peng Xu, Yangfeng Ding, Qing-Sheng Mi, Jianhua Wu, Jun Gu, Yuling Shi

**Affiliations:** 1 Department of Dermatology, Shanghai Skin Disease Hospital, School of Medicine, and; 2 Institute of Psoriasis, School of Medicine, Tongji University, Shanghai, China.; 3 Department of Dermatology, Changhai Hospital, Second Military Medical University, Shanghai, China.; 4 Department of Dermatology, Longhua Hospital, Shanghai University of Traditional Chinese Medicine, Shanghai, China.; 5 Department of Dermatology, Shanghai Tenth People’s Hospital, School of Medicine, Tongji University, Shanghai, China.; 6 Center for Cutaneous Biology and Immunology, Department of Dermatology, and; 7 Immunology Research Program, Henry Ford Cancer Institute, Henry Ford Health System, Detroit, Michigan, USA.

**Keywords:** Dermatology, Inflammation, Dendritic cells

## Abstract

Psoriasis is a chronic, inflammatory skin disease, frequently associated with dyslipidemia. Lipid disturbance in psoriasis affects both circulatory system and cutaneous tissue. Epidermal Langerhans cells (LCs) are tissue-resident DCs that maintain skin immune surveillance and mediate various cutaneous disorders, including psoriasis. However, the role of LCs in psoriasis development and their lipid metabolic alternation remains unclear. Here, we demonstrate that epidermal LCs of psoriasis patients enlarge with longer dendrites and possess elevated IL-23p19 mRNA and a higher level of neutral lipids when compared with normal LCs of healthy individuals. Accordantly, epidermal LCs from imiquimod-induced psoriasis-like dermatitis in mice display overmaturation, enhanced phagocytosis, and excessive secretion of IL-23. Remarkably, these altered immune properties in lesional LCs are tightly correlated with elevated neutral lipid levels. Moreover, the increased lipid content of psoriatic LCs might result from impaired autophagy of lipids. Bulk RNA-Seq analysis identifies dysregulated genes involved in lipid metabolism, autophagy, and immunofunctions in murine LCs. Overall, our data suggest that dysregulated lipid metabolism influences LC immunofunction, which contributes to the development of psoriasis, and therapeutic manipulation of this metabolic process might provide an effective measurement for psoriasis.

## Graphical abstract

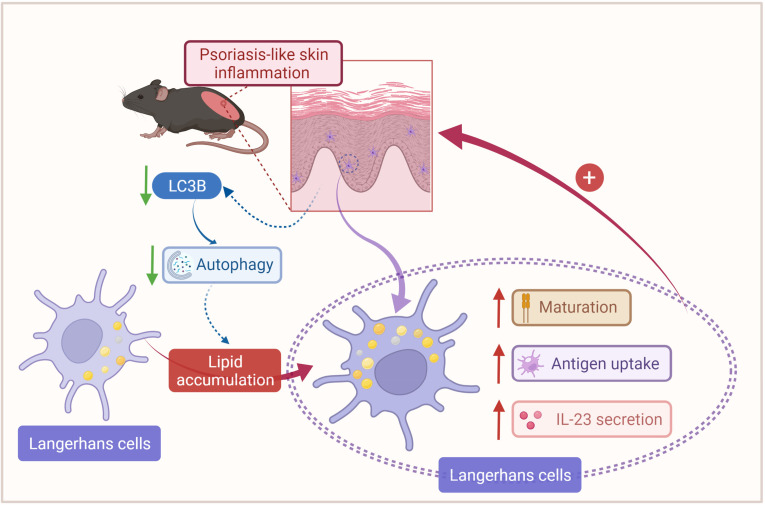

## Introduction

DCs represent a heterogeneous group of potent antigen-presenting cells (APCs) that link innate and adaptive immune responses. Being an exclusive DC subtype within the epidermis, Langerhans cells (LCs) constitute 1%–2% of epidermal cells and characteristically express the C-type lectin langerin, which forms Birbeck granules, the hallmark organelle of epidermal LCs (1). Locating at the first line of defense, immature LCs constantly capture external or intrinsic antigens and subsequently migrate to nearby draining lymph nodes (LNs), where they engage in maturation processes and present engulfed antigens to naïve T cells. Mature LCs secrete cytokines and generate costimulatory signals that influence T cell immune responses (2). In line with their potent immunomodulatory role, LCs participate in the pathogenesis of various skin disorders, including infection, allergy, autoimmunity, and neoplasm.

Psoriasis is a prevalent, chronic, systemic inflammatory disease primarily involving skin tissue and joints, which deleteriously impacts the physical, social, and mental well-being of patients and imposes heavy economic burdens on society (3). Psoriasis is a complex, poly-genetically influenced, immune-mediated disorder triggered by environmental factors such as infection, medication, physical injury, and emotional stress (4). Its immune pathogenesis centers on DC control of T cell polarization, dominated by the IL-23/IL-17 axis, and potentially novel therapies targeted at these cytokines have been proven to be successful in psoriasis treatment (5, 6). However, the exact mechanism of psoriasis remains unclear.

Considerable research effort has focused on the contribution of LCs to the development of psoriasis. Despite inconsistent data regarding the distribution density, epidermal LCs are activated in psoriatic lesions of patients with psoriasis and murine psoriasis-like dermatitis (7). The production of IL-23 and inflammatory chemokines by LCs, and their ability to stimulate T cell immune responses, are considerably enhanced in psoriasis (8–12). The psoriasis-like skin inflammation is generally attenuated in the mice without LCs (7, 11, 13). These findings highlight a proinflammatory role for LCs in the pathogenesis of psoriasis. However, opposite effects have been reported for LCs (14). Thus, the precise role of LCs in psoriasis and the underpinning mechanism requires further exploration.

Psoriasis has well-known correlations with individual components of metabolic syndrome (MS), including dyslipidemia (15). Previous researches demonstrated that lipid disturbance in psoriasis not only referred to systemic dyslipidemia, but also involved altered lipid metabolites within skin tissues (16, 17). An increased level of lipids in the epidermis was uncovered to correlate with the disease severity of psoriasis (18–20). Combined transcriptomic analysis also revealed major changes in lipid pathways in psoriatic skin lesions, indicative of a crucial role for aberrant lipid metabolism in the pathogenesis of psoriasis (21). Recent research demonstrated that lipid metabolism profoundly modulates the physiology of DCs, and altered cellular lipid content in disease states appears to shape the immune responses of DCs (22–24). Thus, we hypothesized that epidermal LCs residing at psoriatic lesions might also possess aberrant lipid profiles, which contribute to its proinflammatory role in the pathogenesis of psoriasis.

Herein, we investigated lipid metabolism in epidermal LCs and its impact on the pathogenesis of psoriasis. For the first time to our knowledge, we uncovered elevated neutral lipids in LCs of both psoriatic lesions from patients and psoriasis-like skin inflammation induced by imiquimod (IMQ), which might primarily cause their overmaturation, enhance phagocytosis, and result in excessive production of IL-23.

## Results

### Epidermal LCs of psoriasis patients possess elevated IL-23p19 mRNA and a higher level of neutral lipids.

**Figure 1:**
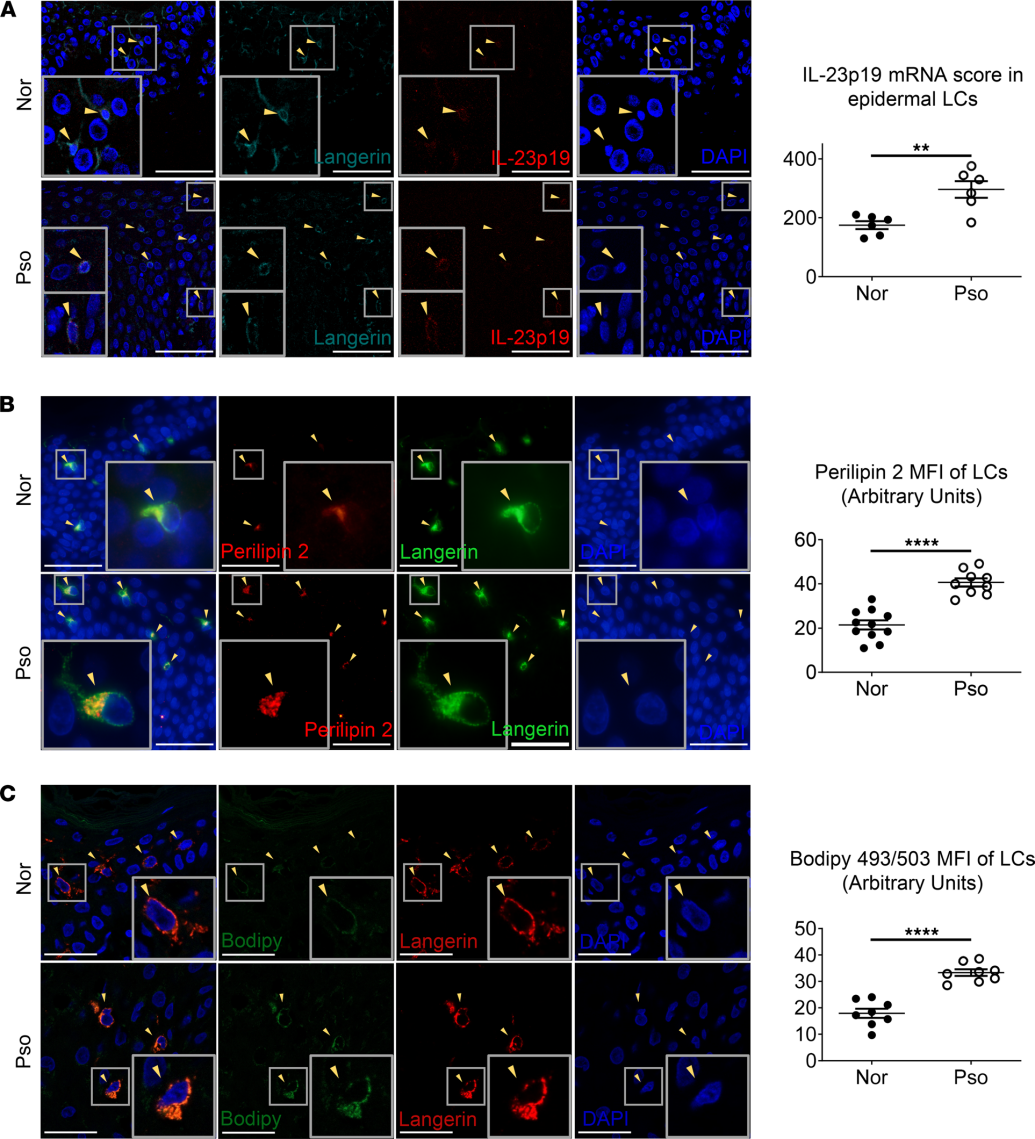
Epidermal LCs of psoriasis patients possess elevated IL-23p19 mRNA and a higher level of neutral lipids. (**A**) Representative in situ hybridization (RNAscope) of IL-23p19 together with immunofluorescence of LCs and IL-23p19 mRNA score in epidermal LCs from FFPE tissue sections of normal human skin and psoriatic lesions (total original magnification, ×400; scale bar : 50 μm; *n* = 12, 2 independent experiments). (**B**) Representative immunofluorescence of perilipin 2 expression and their MFI in LCs from FFPE tissue sections of normal human skin and psoriatic lesions (total original magnification, ×400; scale bar: 50 μm; *n* = 20, 3 independent experiments). (**C**) Representative immunofluorescence of Bodipy 493/503 expression and their MFI in LCs from frozen sections of normal human skin and psoriatic lesions (total original magnification, ×630; scale bar: 30 μm; *n* = 16, 3 independent experiments). Two-tailed Student’s *t* test was performed. The data are presented as mean ± SEM. **P < 0.01, ****P < 0.0001. Arrowheads indicate LCs.

Immunofluorescence and in situ hybridization (RNAscope) were performed to examine the immune properties of epidermal LCs in human psoriatic lesions. Although LC quantity remained largely unaltered — probably due to a dilution by overproliferated keratinocytes, local microenvironment, individual differences, and uneven LC distributions — they became visibly enlarged, with longer dendrites, when compared with normal counterparts of healthy individuals (Supplemental Figure 1; supplemental material available online with this article; https://doi.org/10.1172/jci.insight.150223DS1). The increase in cell volume denoted that epidermal LCs were overmature and immune-activated in psoriasis patients. RNAscope assay further uncovered augmented IL-23p19 mRNA signals in psoriatic LCs when compared with normal LCs (Figure 1A and Supplemental Figure 2). We observed a moderate increase in IL-23p40 mRNA signals in psoriatic LCs, but did not reach statistical significance (Supplemental Figure 3). Since IL-23p40 is a common subunit of IL-12 and IL-23, a IL-23p19 mRNA signal would be the best merit to represent the expression level of IL-23. In line with former reports, our data confirm a proinflammatory role of epidermal LCs in psoriasis development through immune activation and production of IL-17–polarizing cytokines, reminiscent of previous findings for human psoriatic LCs (9, 10).

Recent studies revealed that the lipid content has a strong influence on DC immunofunctions (22, 23). Lipid droplets (LDs) are the major cell organelle for neutral lipid storage (25). Perilipins (PLINs) are the most abundant LD-associated proteins involved in adipogenesis and lipolysis; PLIN2 broadly serves as a marker for LD (26). By calculating the MFI of PLIN2 in each LC (langerin^+^) using ImageJ software (NIH), we detected an upregulation of PLIN2 expression in LCs from psoriatic lesions compared with those from healthy skin (Figure 1B), indicating that psoriatic LCs might have a higher level of neutral lipids than normal counterparts. Further examination of frozen tissue sections from normal human skin and psoriatic lesions with neutral-lipid stain Bodipy 493/505 corroborated that psoriatic LCs had distinctly more neutral lipids than normal LCs (Figure 1C). Therefore, immune-activated epidermal LCs of psoriasis patients are correlated with abnormal lipid profiles.

### LCs display overmaturation, enhanced phagocytosis, and excessive IL-23 secretion in IMQ-induced psoriasis-like dermatitis.

**Figure 2:**
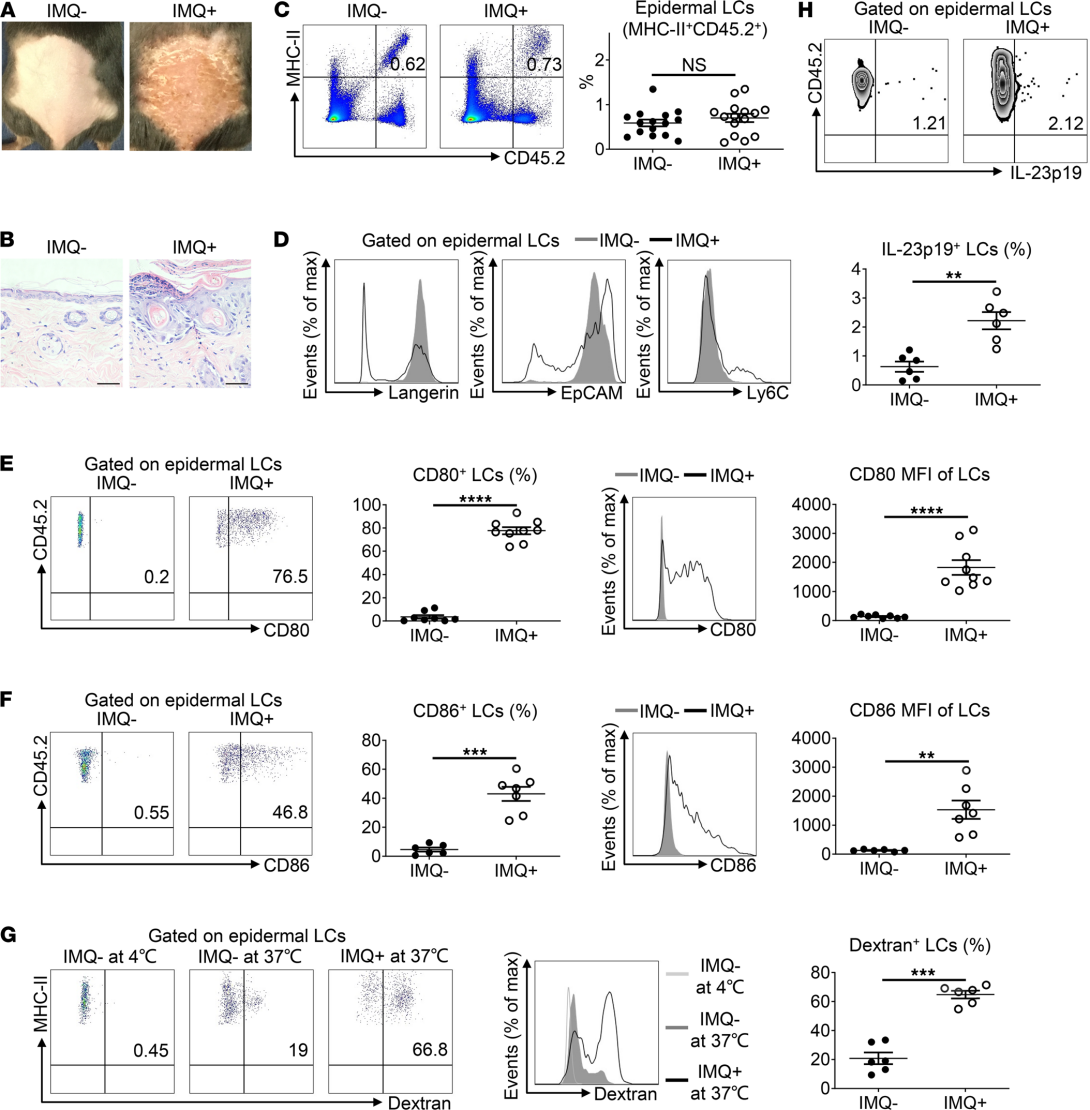
LCs display overmaturation, enhanced phagocytosis, and excessive IL-23 secretion in imiquimod-induced psoriasis-like skin. (**A**) WT C57BL/6J mice were treated daily with IMQ (labeled as IMQ^+^) or control Vaseline cream (labeled as IMQ^–^) application on back skin for 5 consecutive days. (**B**) Representative H&E-stained skin sections (total original magnification, ×400; scale bar: 40 μm) from **A**. (**C**–**F**) Epidermal cells freshly isolated from mouse trunk skin were stained with anti-MHC-II, CD45.2, EpCAM, Ly6C, langerin, CD80, and CD86 Ab and analyzed by flow cytometry. (**C**) Representative FACS analysis and the ratios of epidermal LCs (MHC-II^+^CD45.2^+^) (*n* = 30, 7 independent experiments). (**D**) Representative histograms of langerin, EpCAM, and Ly6C expression in LCs (dark gray filled, IMQ-untreated; black line, IMQ-treated; for langerin, *n* = 12, 3 independent experiments; for EpCAM, *n* = 16, 4 independent experiments; for Ly6C, *n* = 4, 1 independent experiment). (**E** and **F**) Representative FACS analysis, positive ratios, histogram, and MFI of CD80^+^ LCs (**E**) and CD86^+^ LCs (**F**) (dark gray filled, IMQ-untreated; black line, IMQ-treated; for CD80, *n* = 17, 4 independent experiments; for CD86, *n* = 13, 3 independent experiments). (**G**) Freshly isolated epidermal LCs were incubated at 37°C or 4°C (as control) with dextran-FITC for 45 minutes. Representative FACS analysis, histogram, and the percentages of dextran-FITC^+^ epidermal LCs (light gray line, IMQ-untreated at 4°C; dark graey filled, IMQ-untreated at 37°C; black line, IMQ-treated at 37°C; *n* = 12, 3 independent experiments). (**H**) Freshly isolated epidermal cells were in vitro cultured in the presence of Golgi Stop for 4 hours, and they were stained with anti–MHC-II, anti–CD45.2, anti–IL-23p19 Ab and analyzed by flow cytometry. Representative FACS analysis and percentages of IL-23^+^ LCs (*n* = 12, 3 independent experiments). Two-tailed Student’s *t* test was performed. The data are presented as mean ± SEM. **P < 0.01, ***P < 0.001, ****P < 0.0001.

To further explore the potential role of epidermal LCs in the pathogenesis of psoriasis, we next evaluated their disease-associated transformation in an IMQ-induced psoriasis-like dermatitis mouse model. As expected, the application of the TLR7 agonist IMQ resulted in erythematosus, thickened and scaly psoriasis-like dermatitis (Figure 2A), as confirmed by histological observation of hyperkeratosis, dyskeratosis, acanthosis, and dermal inflammatory cell infiltration (Figure 2B). Compared with IMQ-untreated WT C57BL/6J mice (IMQ^–^), no significant change was detected in the LC ratio compared with IMQ-treated mice (IMQ^+^; Figure 2C). Notably, partial LCs expressed a higher level of Ly6C, along with lower levels of langerin and EpCAM (Figure 2D), indicating that monocyte-derived inflamed LCs were repopulated to psoriasis-like skin, which were consistent with previous reports (13, 27).

Under steady states, epidermal LCs are normally immature with limited expression of costimulatory molecules that are indispensable for T cell priming. We noticed a remarkable increase in CD80 and CD86 expression in LCs from IMQ^+^ mice (Figure 2, E and F), in accordance with a previous report (12). However, disease-associated LCs did not significantly upregulate major histocompatibility complex class II (MHC-II) expression (Supplemental Figure 4). In general, epidermal LCs in IMQ-induced psoriasis-like skin was overmature.

Located at the outermost skin layer, LCs efficiently capture and process external or internal antigens, which are essential for mediating immune defenses or tolerance. Upon encountering dextran, a polysaccharide of microbial origin, LCs from IMQ^+^ mice exhibited a significantly higher ratio of dextran-conjugated FITC positivity than LCs from IMQ^–^ mice (Figure 2G), demonstrating that epidermal LCs possess a superior capacity for uptake of antigens under disease conditions.

The IL-23/IL-17 axis plays a fundamental role in the pathogenesis of psoriasis, and LCs are among the main sources of IL-23 (11). Herein, we discovered that normal LCs secreted IL-23 (Supplemental Figure 5), and IMQ application enhanced IL-23 production by LCs (Figure 2H). Moreover, IL-23^+^ LCs had greater expressions of MHC-II and CD80 than IL-23^–^ LCs in both IMQ^–^ and IMQ^+^ mice (Supplemental Figure 6), indicating that LC production of IL-23 was closely associated with their maturation status. Thus, epidermal LCs from IMQ-induced psoriasis-like dermatitis display overmaturation, enhanced phagocytosis, and excessive secretion of IL-23, consistent with our findings for human psoriatic LCs.

### The lipid content of LCs is elevated in IMQ-induced psoriasis-like skin.

**Figure 3:**
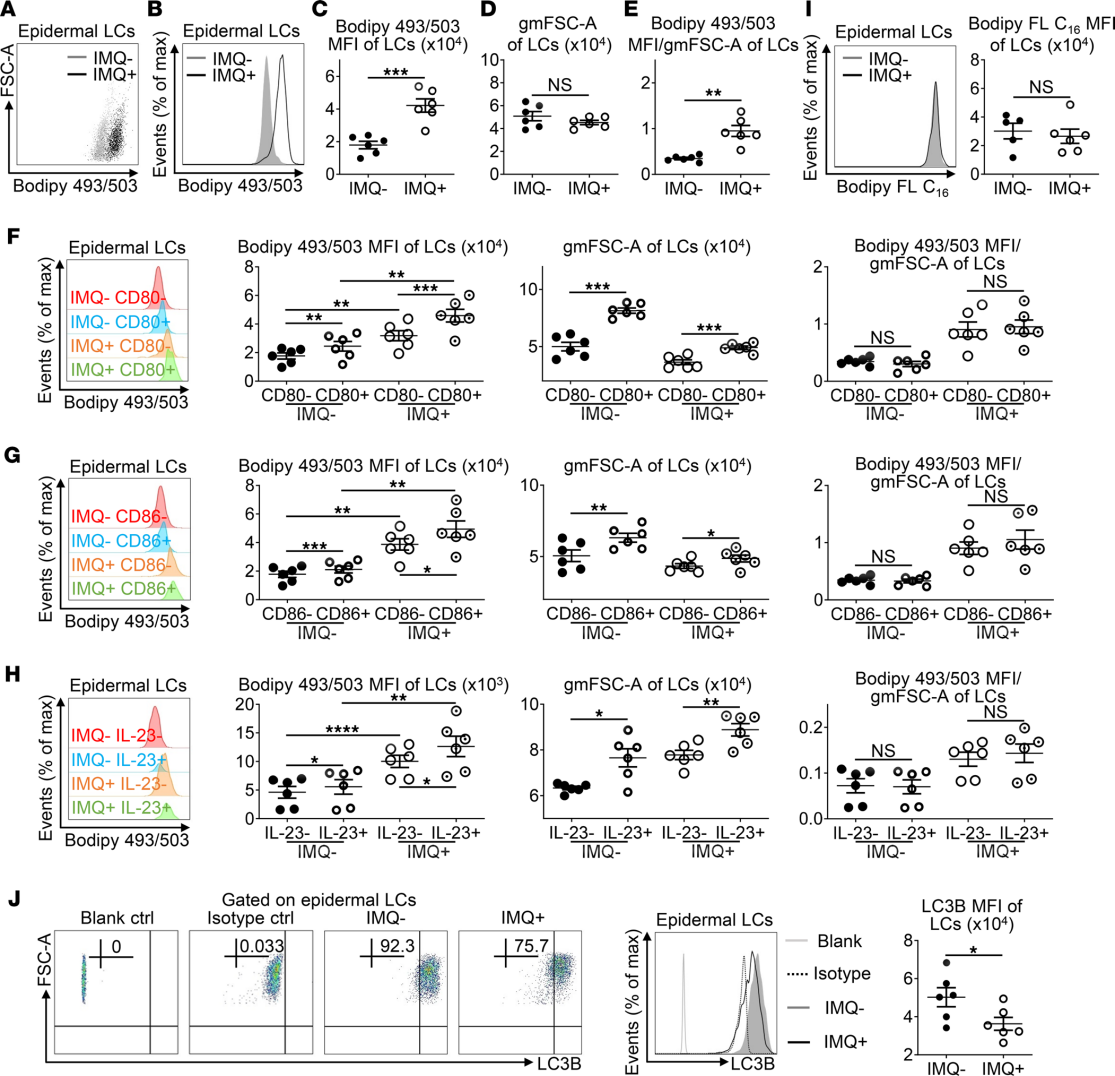
Elevated Langerhans cell lipid content in imiquimod-induced psoriasis-like skin. Mice were treated as in Figure 2. (**A**–**G**) Mouse epidermal cells were stained with anti–MHC-II, anti–CD45.2, anti–CD80, and anti–CD86 Ab and Bodipy 493/503, which were analyzed by flow cytometry (*n* = 12, 3 independent experiments). Representative scatter plots (**A**), histogram (**B**), and MFI (**C**) of Bodipy 493/503 in epidermal LCs. The geometric mean FSC-A (gmFSC-A) (**D**) and the ratio of Bodipy 493/503 MFI to gmFSC-A (**E**) of LCs. Histogram, Bodipy 493/503 MFI, gmFSC-A, and the ratio of Bodipy 493/503 MFI to gmFSC-A of CD80^–^/CD80^+^ LCs (**F**) and CD86^–^/CD86^+^ LCs (**G**) from IMQ^–^ and IMQ^+^ mice. (**H**) Epidermal cells were in vitro cultured with Golgi Stop for 4 hours, and they were stained with anti–MHC-II, anti–CD45.2, and anti–IL-23p19 Ab and Bodipy 493/503, which were analyzed by flow cytometry (*n* = 12, 3 independent experiments). Histogram, Bodipy 493/503 MFI, gmFSC-A, and the ratio of Bodipy 493/503 MFI to gmFSC-A of IL-23^–^ and IL-23^+^ LCs from IMQ^–^ and IMQ^+^ mice. (**I**) Epidermal LCs were incubated at 37°C with Bodipy FL C_16_ (1 μM) for 30 minutes, which were stained with anti–MHC-II and anti–CD45.2 Ab and analyzed by flow cytometry (*n* = 11, 3 independent experiments). Representative histogram and MFI of Bodipy FL C_16_ in epidermal LCs (dark gray filled, IMQ-untreated; black line, IMQ-treated). (**J**) Epidermal LCs were stained with anti–MHC-II and anti–CD45.2 Ab, which were further incubated with anti-LC3B Ab or rabbit IgG isotype control or none (blank control). Representative FACS analysis, histogram, and MFI of LC3B in epidermal LCs (light gray line, blank control; dotted black line, isotype control; dark gray filled, IMQ-untreated; black line, IMQ-treated; *n* = 12, 3 independent experiments). Two-tailed Student’s *t* test was performed. In (**F–H**), tests were considered significant with *P* < 0.05 after multiple testing adjustments by the FDR method. The data are presented as mean ± SEM. *P < 0.05, **P < 0.01, ***P < 0.001, ****P < 0.0001.

To test if the cellular lipid content in LCs was also altered in psoriasis-like dermatitis, epidermal cells were incubated with Bodipy 493/503. Remarkably, the median fluorescence intensity (MFI) of Bodipy 493/503 in lesional LCs was approximately twice that of normal LCs (Figure 3, A–C). To eliminate the possibility that cell volume might determine the levels of intracellular substances, we utilized geometric mean forward scatter-area (FSC-A) (gmFSC-A) as an indicator of cell volume. Since the gmFSC-A of LCs was comparable between IMQ^–^ and IMQ^+^ mice (Figure 3D), the ratio of Bodipy 493/503 MFI to gmFSC-A from lesional LCs was still considerably higher than normal LCs (Figure 3E), confirming an increase in the lipid content of epidermal LCs from IMQ-induced psoriasis-like skin tissues.

Further analysis demonstrated that mature LCs (CD80^+^ or CD86^+^) possessed a higher lipid content than immature LCs in a cell volume–dependent manner (Figure 3, F and G), suggesting that the process of LC maturation correlated with increased lipid content. Since the production of IL-23 by epidermal LCs is associated with maturation status, IL-23^+^ LCs also contained more lipid than IL-23^–^ LCs in both IMQ^–^ and IMQ^+^ mice (Figure 3H). Together, these results imply that cellular lipids might substantially influence LC maturation and secretion of IL-23.

To investigate whether the high lipid content of lesional LCs resulted from enhanced phagocytosis of microenvironmental lipids, epidermal cells were incubated with Bodipy FL C_16_ fatty acid (FA). LCs achieved maximum uptake of Bodipy FL C_16_ between 30 and 45 minutes (Supplemental Figure 7). Although LCs from IMQ-induced psoriasis-like skin displayed a greater capacity to engulf antigens, the Bodipy FL C_16_ MFI of lesional LCs was equivalent to that of normal LCs after a 30-minute incubation with Bodipy FL C_16_ (Figure 3I). Thus, the elevated lipid content in epidermal LCs from IMQ-induced psoriasis-like skin was not caused by heightened uptake of microenvironmental lipids.

Autophagy, an evolutionarily conserved catabolic program that degrades intracellular materials, was recently shown to be a vital regulator of lipid metabolism (28). To explore whether higher lipid levels in lesional LCs were caused by diminished autophagy of lipids stored in LDs, we assessed the expression of autophagic marker microtube-associated protein 1 light chain 3B (LC3B) in epidermal LCs (29). We found that LCs from IMQ^+^ mice expressed lower levels of LC3B compared with LCs from IMQ^–^ mice (Figure 3J), implying that autophagy might be hampered in LCs after IMQ application. To further explore whether autophagy was likewise affected in epidermal LCs of psoriasis patients, we performed an immunofluorescence assay and found that psoriatic LCs had significantly fewer LC3B puncta than normal counterparts (Supplemental Figure 8), denoting that LCs from psoriatic lesions had decreased autophagy compared with healthy LCs.

In a nutshell, epidermal LCs from human psoriatic lesions and psoriasis-like murine skin tissues display similar immune modifications regarding activation status, IL-23 secretion, and lipid accumulation. Specifically, the increased lipid content of epidermal LCs might result from impaired autophagy of lipids rather than altered lipid engulfment. Moreover, cellular lipids might affect the maturation of LCs and their secretion of IL-23.

### Inhibition of FA synthesis alters the immunofunctions of epidermal LCs.

**Figure 4:**
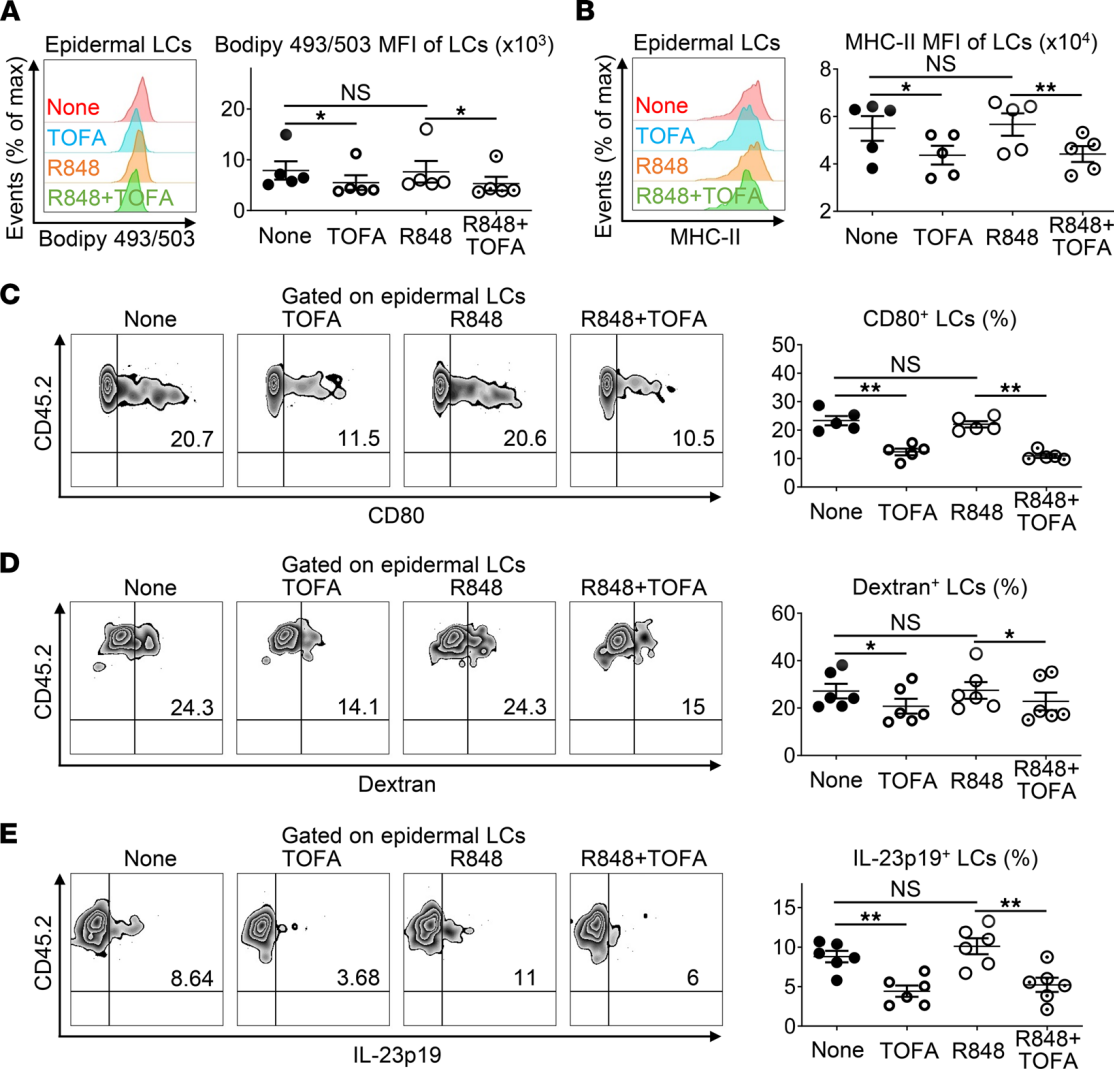
Inhibition of fatty acid synthesis alters the immunofunctions of epidermal LCs. (**A**–**E**) Epidermal cells freshly isolated from WT C57BL/6J mice were in vitro cultured with or without Resiquimod (R848, 2 μg/mL) or/and TOFA (10 μg/mL) for 18 hours (**A**–**C**) or 24 hours (**D** and **E**) with or without Golgi Stop for the last 4 hours. (**A**–**C**) Cells were stained with anti–MHC-II, anti–CD45.2, anti–CD80 Ab and Bodipy 493/503, which were analyzed by flow cytometry (*n* = 20, 3 independent experiments). Histogram and MFI of Bodipy 493/503 (**A**) and MHC-II (**B**) in epidermal LCs. (**C**) Representative FACS analysis and the ratios of CD80^+^ LCs. (**D**) Cells were incubated with dextran-FITC at 37°C for 45 minutes and stained with anti–MHC-II and anti–CD45.2. Representative FACS analysis and the ratios of dextran^+^ LCs (*n* = 24, 3 independent experiments). (**E**) Cells were stained with anti-MHC-II, anti–CD45.2 and anti–IL-23p19. Representative FACS analysis and the ratios of IL-23p19^+^ LCs (*n* = 24, 3 independent experiments). Two-tailed Student’s *t* test was performed. Tests were considered significant with *P* < 0.05 after multiple testing adjustments by the FDR method. The data are presented as mean ± SEM. *P < 0.05, **P < 0.01.

To further determine the impact of lipid content on LC immune functions, 5-tetradecyloxy-2-furoic acid (TOFA), an allosteric inhibitor of acetyl-CoA-carboxylase-α (ACCα), was employed to decrease FA synthesis in epidermal LCs. As depicted in Figure 4A, even though resiquimod (R848), a TLR7 and TLR8 agonist that mimics IMQ, failed to raise the cellular lipid content, in vitro stimulation with TOFA significantly reduced neutral lipid levels in LCs. Concomitantly, expression of mature markers MHC-II and CD80, the phagocytic capacity of dextran-conjugated FITC, and the secretion of IL-23 were all considerably diminished in TOFA-treated LCs containing lower lipid levels, whereas the immune traits of R848-stimulated LCs — harboring a lipid content equivalent to untreated controls — remained unaltered (Figure 4, B–E), demonstrating that intracellular lipid levels are essentially correlated with the immunofunctions of epidermal LCs. Remarkably, psoriasis area and severity index (PASI) scores showed that s.c. administration of TOFA alleviated IMQ-induced psoriasis-like skin inflammation (Supplemental Figure 9, A–D). PASI scores were comprised of erythema (scores 0–4), infiltration (scores 0–4), and desquamation (scores 0–4) scores. The application of TOFA altered skin thicknesses and histologic features. Concurrently, cellular lipid increases in LCs from IMQ^+^ mice were moderately compromised by TOFA application (Supplemental Figure 9E), which further indicates an enormous impact of lipid levels on LC immunofunctions in IMQ-induced psoriatic dermatitis.

### Lipidomic analysis of epidermal LCs.

**Figure 5:**
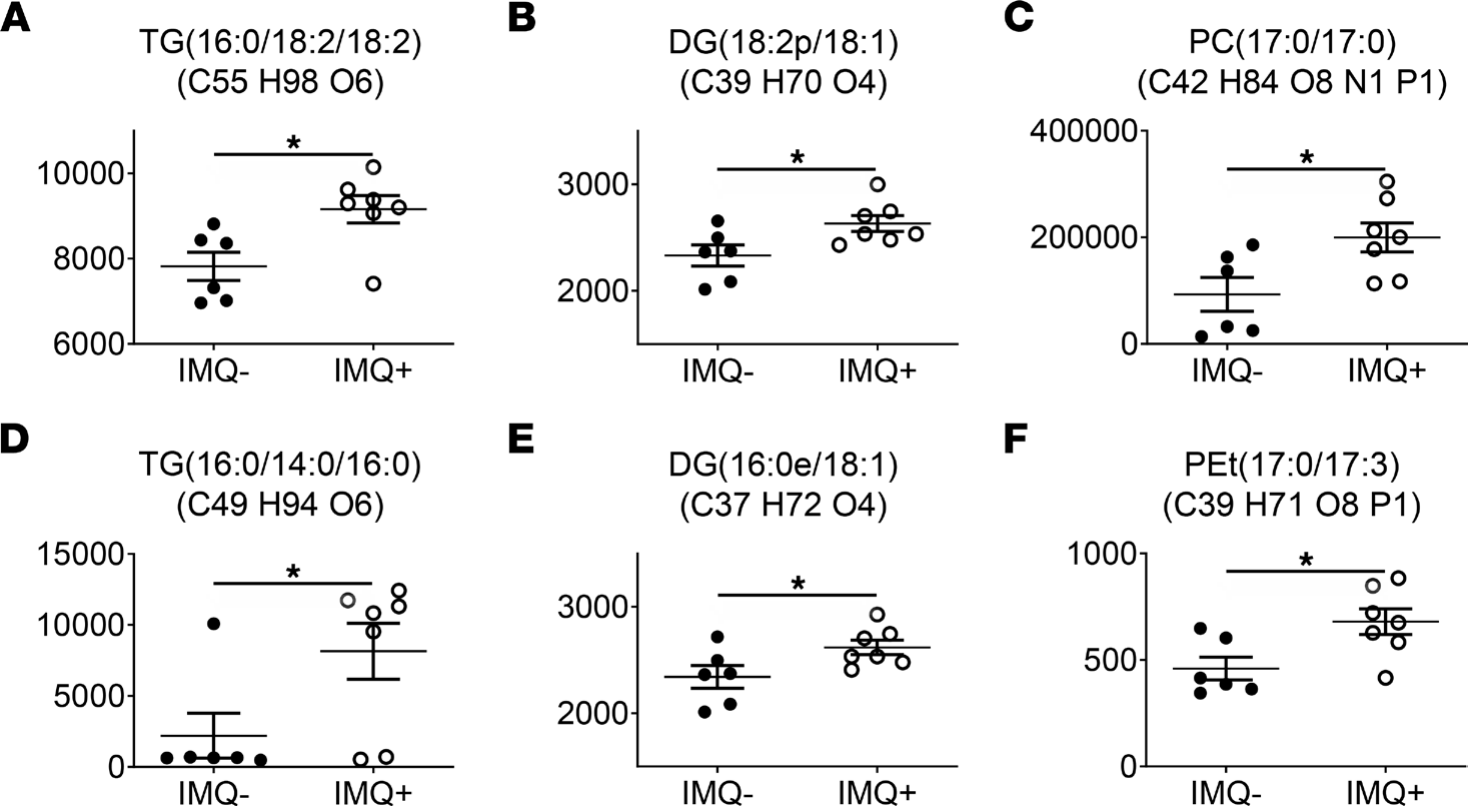
Lipidomic analysis of epidermal LCs. Freshly isolated epidermal cells from IMQ^–^ and IMQ^+^ mice underwent dead-cell removal and LC enrichment using microbeads. Epidermal LCs were freshly sorted from LC-enriched epidermal cell suspensions by flow cytometry. Lipids were extracted from the separated LCs and tested by LC-MS analysis. (**A**–**F**) The peak areas of differentially expressed lipid metabolites between normal and disease-associated LCs. TG, triglyceride; DG, diglyceride; PC, phosphatidylcholine; PEt, phosphatidyl ethanol. *n* = 13. Two-tailed Student’s *t* test was performed. The data are presented as mean ± SEM. *P < 0.05.

To identify the lipid metabolites responsible for the elevated lipid content in lesional LCs, epidermal LCs were freshly isolated from IMQ^–^ and IMQ^+^ mice (Supplemental Figure 10) and subjected to lipid profiling by liquid chromatography–mass spectrometry (LC-MS) analysis. Excellent reproducibility of the retention time (RT) was observed based on overlap of total ion chromatograms for quality control samples (Supplemental Figure 11). Among the ~300 lipid metabolites detected in the different LCs, the cellular content of 6 lipids was significantly higher in disease-correlated LCs compared with normal equivalents, including 2 triglycerides (TGs), 2 diglycerides (DGs), 1 phosphatidylcholine (PC), and 1 phosphatidyl ethanol (PEt; Figure 5). These increases in TG, DG, PC and PEt might directly contribute to elevated lipid levels in epidermal LCs from IMQ-induced psoriasis-like cutaneous lesions.

### Gene expression profiling of epidermal LCs.

**Figure 6:**
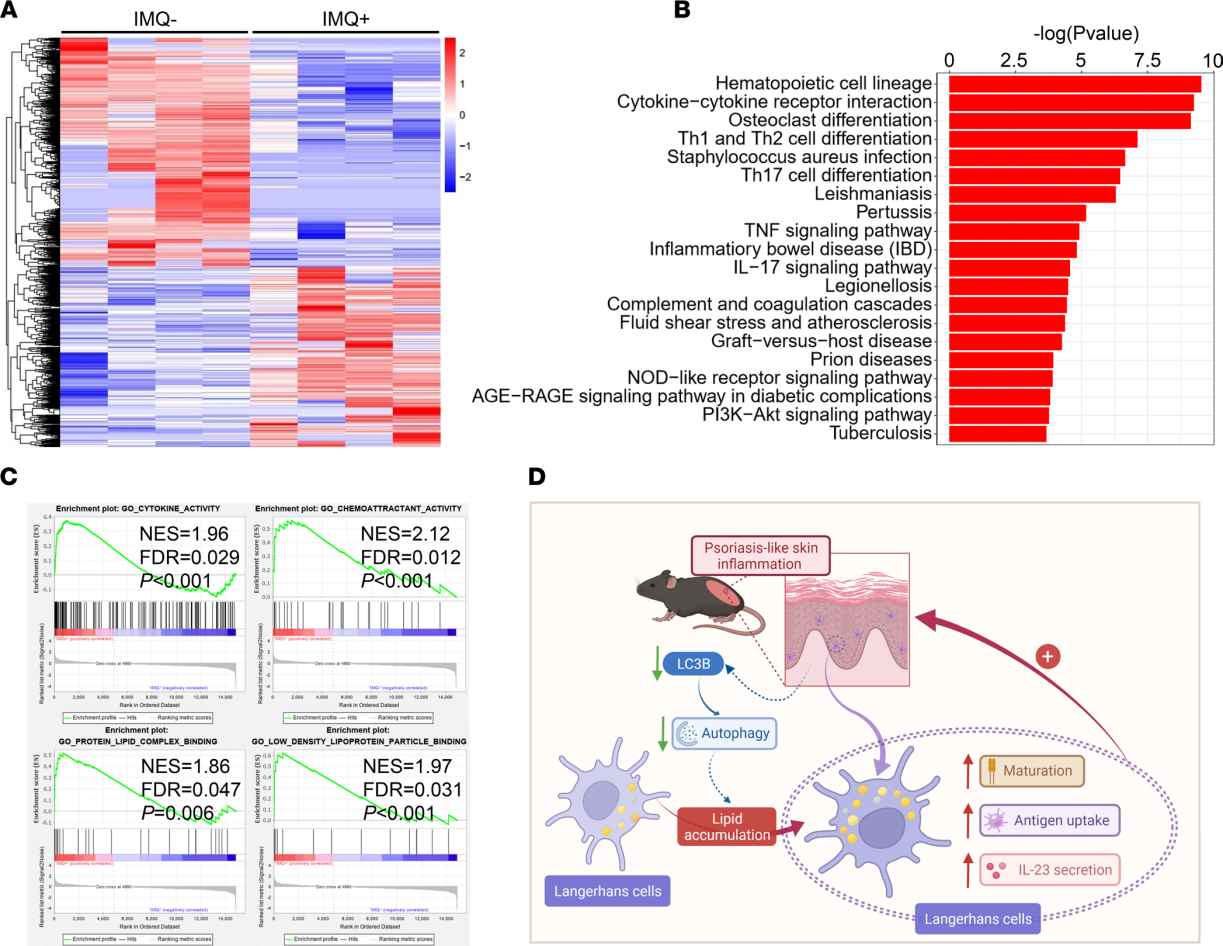
Gene expression profile of epidermal LCs. (**A**–**C**) Epidermal LCs were freshly sorted from IMQ^–^ and IMQ^+^ mice and underwent low-input mRNA-Seq analysis. Heatmap clustering analysis (**A**), Kyoto Encyclopedia of Genes and Genomes (KEGG) pathway analysis (**B**) and Gene Set Enrichment Analysis (GSEA) (**C**) of RNA-Seq data. (**D**) Possible functional mode of the lipid metabolic alternations of epidermal LCs in psoriasis-like skin inflammation.

To explore the potential mechanisms contributing to the altered nature of LCs within psoriasis-like skin lesions, freshly separated epidermal LCs from IMQ^–^ and IMQ^+^ mice were subjected to low-input mRNA-Seq (GSE162274, Gene Expression Omnibus [GEO]). Clustering analysis of differentially expressed genes (DEGs) was performed by heatmap analysis, which revealed differences in the transcriptome of normal and disease-associated LCs (Figure 6A and Supplemental Table 1). Kyoto Encyclopedia of Genes and Genomes (KEGG) pathway analysis implied that lesional LCs were highly enriched in several inflammatory signaling pathways, including cytokine-cytokine receptor interaction; Th1, Th2, and Th17 cell differentiation; TNF signaling and IL-17 signaling pathways; complement and coagulation cascades; and NOD-like receptor signaling and PI3K/Akt signaling pathways (Figure 6B). Gene set enrichment analysis (GSEA) plots revealed enrichment of cytokine activity, chemoattractant activity, protein lipid complex binding, and low-density lipoprotein particle–binding gene sets in LCs from IMQ^+^ mice compared with IMQ^–^ mice (Figure 6C and Supplemental Table 2). Together, these results confirmed a proinflammatory role for epidermal LCs in the pathogenesis of psoriasis. Our findings, summarized in Figure 6D, show that the inflammatory microenvironment of IMQ-induced psoriasis-like dermatitis might impair the autophagic process of epidermal LCs and cause neutral lipid accumulation, which would result in LC immune activation and form an amplifying loop of inflammation.

## Discussion

Despite their versatile immunofunctions, previous reports and our results herein indicate that epidermal LCs might play a proinflammatory role in the pathogenesis of psoriasis. In psoriasis patients, epidermal LCs appeared larger than normal counterparts with extended dendrites, implying that they were activated and became mature (7). Epidermal LC migration was inhibited in psoriasis patients, probably due to an alteration of the keratinocyte secretome induced by IL-17 (30–32). Transcriptional profiling of psoriatic LCs revealed expression of attracting chemokines such as C-X-C motif ligand 1 (CXCL1) and CXCL10, as well as inﬂammatory chemokines including chemokine ligand 18 (CCL18) and CCL20 (8). Furthermore, a significantly higher ratio of epidermal LCs in lesional and perilesional tissues produced IL-23 compared with LCs from healthy donors (7, 9). Even after treatment with TNF inhibitors, epidermal LCs retained elevated IL-23 expression (10). The proinflammatory features of detained LCs in psoriatic lesions imply that epidermal LCs might steer skin-resident memory T cells and sustain local inflammation in psoriasis (33). Likewise, results from animal research resemble findings in humans. The absence of LCs in multiple transgenic mice significantly alleviated psoriasis-like skin inflammation (7, 11, 13). Epidermal LCs are the major DC subset to generate IL-23, and they promote IL-17 secretion by dermal αβ and γδ T cells in IMQ-treated mice (11, 34). In this process, p38α signaling probably participates in IL-23 production by LCs (34). Consistently, we observed that epidermal LCs in IMQ-induced psoriasis-like dermatitis upregulated the expression of costimulatory molecules and oversecretion of IL-23. We have also observed that epidermal LCs of psoriasis patients enlarge with longer dendrites and possess elevated IL-23p19 mRNA. Moreover, transcriptional analysis of murine lesional LCs implied immune activation and a promotive role in skin inflammation. Nevertheless, paradoxical results have also been reported. Epidermal LCs from psoriatic lesions express higher mRNA levels of several tolerogenic factors, such as programmed death-ligand 1 (PD-L1), PD-L2, and indoleamine 2,3-dioxygenase 1 (IDO-1) (10). Meanwhile, in *Jun^fl/fl^ JunB^fl/fl^ K5cre-ERT* mice, LCs elicited antiinflammatory activity during active psoriatic disease (14). These discrepancies might result from the negative feedback of inflammation in LCs and/or differences in mouse genetic backgrounds. Collectively, our results further support a proinflammatory role for epidermal LCs in psoriasis.

The earliest study on lipid accumulation in DCs was published in 2005, which uncovered that BM-derived DCs became “lacy” cells packed with large amounts of fat, and this transformation was promoted by antigen encounter along with cytokine stimulation (35). Later, tumor-associated lipid-overloaded DCs were reported from individuals with head and neck cancer (HNC) and EL-4 tumor–bearing mice, and normalization of lipid abundance with ACC inhibition restored the immunofunctions of DCs (22). Similar results were acquired for peripheral myeloid DCs from patients with lung cancers (36) and murine DCs from radiation-induced thymic lymphomas (24). Unlike tumor-associated lipid-laden DCs eliciting antiinflammatory functions, a unique lipid-based dichotomy was observed in hepatic DCs; lipid-rich DCs were immunogenic, whereas DCs with low levels of lipids mediated immune tolerance (23). Likewise, the absence of ATP-binding cassette transporters A1 and G1 (ABCA1/G1) in DCs results in unesterified total cholesterol (TCH) accumulation, inflammasome activation, and enhanced inflammatory cytokine secretion, which promotes T cell activation and polarization of Th1 and Th17 cells (37). Herein, we demonstrated that epidermal LCs in the skin lesions of psoriasis patients and psoriasis-like skin inflammation accumulated cellular lipids, and this correlated with their immune activation and oversecretion of IL-23. Divergent lipid-triggering immune responses might result from differences in the type of DC subset, type of labile lipids, and cytokine microenvironment. While TG was abundant in tumor-associated tolerogenic DCs, increases in TCH or a mixture of TG, DG, PC, and PEt were observed in immunogenic DCs (22, 24, 37). Although we did not observe any increase in neutral lipids in LCs in vitro following stimulation by IL-17A (our unpublished observations), which was inconsistent with human monocyte-derived DCs (38), difference in the types and amounts of cytokines in different tissues under healthy and diseased states might influence DC immunofunctions.

The accumulation of lipids in DCs has been postulated to result from aberrant expressions of scavenger receptor A, lipoprotein lipase (LPL), FA-binding protein 4 (FABP4), and ABCA1/G1 (22, 24, 37). Scavenger receptor A mediates the influx of lipids, which is involved in foam cell formation. LPL catalyzes the hydrolysis of TG and helps cells to acquire FAs (39). FABPs are a family of conserved proteins that bind medium- to long-chain FAs to assist their uptake, transport, and metabolism (40). We discovered that diminished autophagy of lipids rather than altered lipid engulfment might lead to lipid accumulation in psoriatic LCs. Our transcriptional data consistently reveal increased *Plin2* and lower *Lc3a* mRNA expression levels (Supplemental Figure 12). Additionally, we identified multiple potential factors that might also contribute to elevated lipid levels in disease-associated LCs through RNA-Seq analysis, including upregulation of macrophage scavenger receptor 1 (*Msr1*) and *Fabp5*, and downregulation of *Abca9* (Supplemental Figure 12); FABP5 is a cytoplasmic long-chain FA–binding protein that participates in multiple physiological processes, including mitochondrial FA β-oxidation and transport, membrane phospholipid synthesis, and lipid metabolism (41). Previous studies demonstrated that FABP5 is tightly involved in the development of psoriasis through regulating keratinocyte differentiation and mediating systemic metabolic disturbances (42, 43). Upregulated FABP5 expressions in epidermal LCs of human psoriatic lesions and psoriasis-like murine skin tissues were verified by immunohistofluorescence analysis (Supplemental Figure 13). Future studies utilizing LC-specific FABP5-KO or overexpressed mice are required to unveil the role of FABP5 in the lipid metabolic change of psoriatic LCs. Since in vitro R848 coculture failed to induce elevated lipids in LCs, TLR7 stimulation does not initiate lipid accumulation. Thus, the detailed mechanism by which lipid levels are elevated in LCs within psoriasis-like dermatitis requires further investigation.

Recent research has advanced our understanding of why lipid accumulation suppresses the function of tumor-associated DCs (44, 45). In tumor-bearing hosts, DCs accumulate LDs containing oxidized lipids, which trapped chaperone heat shock protein 70 (HSP70) (44). This interaction prohibits the translocation of peptide-loaded MHC-I (pMHC-I) molecules to the cell surface, which compromised the ability of tumor-associated DC to stimulate adequate CD8^+^ T cell responses (44). On the other hand, defects in LD formation in splenic and BM-derived DCs under steady states may hamper cross-presentation of engulfed antigens to CD8^+^ T cells — but not antigen presentation to CD4^+^ T cells (46). The delicate regulation of LD formation and antigen presentation in DCs complicates investigation of the underlying mechanism. Based on prior research on LCs and our transcriptional analysis, we predict that lipids accumulated in LCs might activate PI3K/Akt, p38 MAPK, or NF-κB signaling pathways, which may lead to aberrant immune functions, but this hypothesis awaits further investigation. Previous research on LCs has been hampered due to difficulties in obtaining enough material, poor availability of appropriate cell lines, and a lack of LC-specific transgenic mice for that; although langerin-CRE mice are being widely utilized, langerin is not uniquely expressed in LCs. With rapid technical advances, we anticipate overcoming these difficulties and determining the mechanism of this minor but important cell population.

Even though DC activation initiates the involvement of the IL-23/IL-17 axis in the pathogenesis of psoriasis, the exact process remains obscure, and the antigen recognized by DCs in psoriasis has long been unidentified. Recent evidence demonstrated that, in psoriatic lesions, epidermal LCs utilize CD1a to present neolipid antigens to CD1a-reactive IL-17–producing T cells (47). Herein, we revealed that lesional LCs were abnormally mature and simultaneously displayed a heightened capacity for phagocytosis, unlike naturally mature LCs with diminished antigen uptake. Since macropinocytosis is shut down upon spontaneous DC maturation, receptor-mediated endocytosis might operate in LCs from psoriasis-like cutaneous lesions (48). The significance of this conversion of phagocytosis and its impact on antigen-presentation requires further exploration.

In conclusion, we observed overmaturation, enhanced phagocytosis, and IL-23 oversecretion of psoriatic LCs. Remarkably, disease-associated LCs possessed elevated levels of neutral lipids, comprising TG, DG, PEt, and PC, and this was tightly correlated with their immune properties. Consistently, transcriptional analysis of murine disease–associated LCs identified expression of multiple genes involved in lipid metabolism and autophagy, and associated immunofunctions were dysregulated accordingly. Our results highlight an essential role for lipid metabolism in LC immunofunctions and its contribution in the pathogenesis of psoriasis.

## Methods

### Animals.

C57BL/6J mice were purchased from Lingchang Biotech and were bred and maintained in specific pathogen–free (SPF) units with controlled temperature (22°C ± 2°C), relative humidity (50% ± 5%), artificial light (12-hour light/dark cycle), and free access to food/water in the animal facilities of Tongji University. Age- and sex-matched mice at 8–10 weeks of age were randomly used for all experiments. Handling of mice and experimental procedures were in accordance with the requirements of Animal Care and Use Committee of Shanghai Tongji University.

### IMQ-induced psoriasis-like skin inflammation.

Mice received a daily topical dose of 62.5 mg of commercially available IMQ cream (5%; H20030128, Sichuan Med-Shine Pharmaceutical) or control Vaseline (VAS) onto a shaved back patch for 5 consecutive days. TOFA (T6575, MilliporeSigma) or phosphate-buffered saline (PBS; SH30256.01B, Hyclone) was administered s.c. at a dose of 5 mg/kg body weight 1 day before the application of VAS or IMQ cream, and administration continued for 6 successive days. Mouse skin lesions were assessed by PASI scores, which were comprised of erythema (scores 0–4), infiltration (scores 0–4), and desquamation (scores 0–4) scores. Skin thicknesses were measured by vernier callipers. Paraffin-embedded skin specimens were prepared using routine methods, and sections were stained with H&E.

### Epidermal single-cell suspension preparation.

Mouse skin was incubated with dispase II (3 mg/mL; D4693, MilliporeSigma) for 1 hour at 37°C after s.c. adipose tissue was scraped off. The epidermal sheet was peeled from the dermis, cut into small pieces, and digested at 37°C in PBS containing trypsin-EDTA (0.05%; 25200072, Thermo Fisher Scientific) and DNase (0.01%; B002004, Diamond) for 15 minutes or complete culture medium containing DNase (0.01%) for 1 hour by gently shaking. Complete culture medium was RPMI 1640 (11875119, Thermo Fisher Scientific) supplemented with heat-inactivated FBS (10%; SH30084.03, Hyclone), 2-mercaptoethanol (5.5 μM; 21985023, Thermo Fisher Scientific), HEPES buffer (20 mM; 15630080, Thermo Fisher Scientific), sodium pyruvate (1 mM; 11360070, Thermo Fisher Scientific), nonessential amino acids (11140050, Thermo Fisher Scientific), penicillin G (100 U/mL; B540733, Sangon), streptomycin sulphate (100 μg/mL; B540733, Sangon), and amphotericin B (2.5 μg/mL; B540733, Sangon). The epidermal single-cell suspension was harvested by passing through a 40 μm cell strainer (CSS-010-040, Jet Biofil).

### Phagocytosis assay.

To determine the capacity of LCs to engulf foreign antigens, freshly isolated epidermal cells were incubated with dextran-fluorescein isothiocyanate (dextran-FITC, 0.025%; FD4, MilliporeSigma) for 45 minutes at 37°C or 4°C. To evaluate the ability of LCs to uptake FAs, epidermal cells were incubated with Bodipy FL C_16_ (1 μM; D3821, Invitrogen) for 30 minutes at 37°C. Subsequently, cells were rinsed with PBS and stained with antibodies.

### Cell culture.

To induce in vitro maturation of LCs, freshly separated epidermal cells were cultured in complete culture medium at 37°C for 60 hours without media change. For intracellular cytokine staining, cells were incubated with or without Resiquimod (2 μg/mL; SML0196, Sigma) and/or TOFA (10 μg/mL; T6575, Sigma) for 4, 18, or 24 hours, with or without Golgi Stop (554724, BD Biosciences).

### Flow cytometric analysis.

Single-cell suspensions were pretreated with anti-CD16/CD32 Ab (clone 2.4G2; 553141, BD Biosciences) for 10 minutes at 4°C. To discriminate viable cells from nonviable cells, cells were stained with fixable viability stain (564406, BD Biosciences) for 10 minutes at room temperature. For analysis of surface markers, cells were stained with anti–mouse MHC-II Ab (clone M5/114; 562363, BD Biosciences), anti–mouse CD45.2 Ab (clone 104; 109832, BioLegend), anti–mouse CD80 Ab (clone 16-10A1; 104708, BioLegend), and anti–mouse CD86 Ab (clone GL-1; 105012, BioLegend) for 30 minutes at 4°C in the dark. For intracellular staining, cells were fixed and permeabilized with BD Cytofix/Cytoperm (51-2090KZ, BD Biosciences) and stained with anti–mouse langerin Ab (clone 4C7; 144204, BioLegend), anti–mouse IL-23p19 Ab (clone N71-1183; 565317, BD Biosciences), its isotype control anti–mouse Rat IgG1κ (clone R3-34; 557731, BD Biosciences), and incubated for 30 minutes at 4°C. For LC3B labeling, permeabilized cells were incubated with anti-LC3B polyclonal Ab (PA1-46286, Invitrogen) or anti–mouse rabbit IgG isotype control (10500C, Invitrogen) for 40 minutes at room temperature, which were then labeled with Alexa Fluor 647 goat anti–rabbit IgG (A-21244, Invitrogen) for 30 minutes at room temperature. To evaluate cellular lipid level, cells were suspended in Bodipy 493/503 (5 μM; D3922, Invitrogen) for 15 minutes at 37°C. Finally, cells were assayed using a BD LSRFortessa Cytometer and analyzed with FlowJo software (BD Biosciences).

### Immunohistofluorescence staining.

Immunohistofluorescence staining was performed on 4 μm formalin-fixed, paraffin-embedded (FFPE) tissue sections of normal human skin, psoriatic lesions, and mouse skin specimens. After deparaffinization and autofluorescence reduction, sections were heated in Tris-EDTA buffer (pH 9.0, G1203, Servicebio) for 20 minutes to reverse formaldehyde cross-links, followed by blocking with PBS containing 1% BSA (36101ES76, Yeasen Biotech) for 1 hour at room temperature. Sections were incubated with rabbit monoclonal anti-langerin Ab (clone D9H7R; 13650, CST) at a dilution of 1:50 overnight at 37°C, and then labeled with Alexa Fluor 488 goat anti–rabbit IgG (A-11008, Invitrogen) or Alexa Fluor 555 goat anti–rabbit IgG (A-21429, Invitrogen) at a dilution of 1:500 for 2 hours at room temperature. For PLIN2 staining, sections were incubated with mouse monoclonal anti-PLIN2 Ab (clone 2C5A3; ab181463, Abcam) at a dilution of 1:50 overnight at 37°C, and they were then labeled with Alexa Fluor 555 goat anti–mouse IgG (A-21422, Invitrogen) at a dilution of 1:500 for 2 hours at room temperature. Costaining of rabbit monoclonal anti-FABP5 Ab (clone D1A7T; 13650, CST) and rabbit monoclonal anti-langerin Ab (clone D9H7R; 13650, CST) or rat monoclonal anti-langerin Ab (clone 929F3.01; DDX0362P, Origene) was conducted using a Three-color Fluorescence Kit (RC0086-23, Recordbio Biological Technology) based on tyramide signal amplification technology according to the manufacture’s instruction. Finally, tissue sections were counterstained with DAPI (422801, BioLegend) and mounted with ProLong Gold Antifade Mountant (P36930, Invitrogen).

Immunohistofluorescence staining was also performed on 8μm frozen, optimal cutting temperature compound–embedded (OCT-embedded) tissue sections. Sections were fixed by PBS containing 3.7% formalin for 1 hour at room temperature and were blocked by PBS containing 1% BSA (36101ES76, Yeasen Biotech) for 1 hour at room temperature. For Bodipy 493/505 staining, sections were firstly stained by anti-langerin Ab (clone D9H7R; 13650, CST) and then stained with 1 μL/mL Bodipy 493/503 for 30 minutes at room temperature. For LC3B staining, sections were incubated with rabbit polyclonal anti-LC3B Ab (PA1-46286, Invitrogen) at a dilution of 1:200 overnight at 4°C, and it was then labeled with Alexa Fluor 555 goat anti–rabbit IgG (A-21429, Invitrogen) at a dilution of 1:500 for 1 hour at room temperature. Finally, tissue sections were counterstained with DAPI (422801, BioLegend) and mounted with ProLong Gold Antifade Mountant (P36930, Invitrogen).

Images were captured on an BX53 Upright Fluorescence Microscope (Olympus), a CX33 Upright Fluorescence Microscope (Yuehe Biological Technology), or a LSM900 Confocal Microscope (Carl Zeiss) and were processed using open-source ImageJ software (NIH). A section of 1 psoriatic lesion and its adjacent nonpsoriatic skin tissue from a psoriasis patient was scanned with a Pannoramic MIDI scanner (3DHISTECH) and processed using CaseViewer software (3DHISTECH).

### RNAscope in situ hybridization combined with immunofluorescence.

RNAscope assay was performed on 4 μm FFPE sections using the Multiplex Fluorescent Reagent Kit v2 (323100, Advanced Cell Diagnostics [ACD]) according to the manufacturer’s instructions. Briefly, FFPE sections were dewaxed with xylene, pretreated with hydrogen peroxide (322335, ACD), target retrieved in Target Retrieval Reagents (322001, ACD), and permeabilized by Protease Plus (322331, ACD). Hybridization of the target probe Hs-IL23A (562851-C2, ACD) or Hs-IL12B (319551-C2, ACD) was performed by incubation at 40°C for 2 hours, followed by standard signal amplification steps. Probes Hs-PPIB human peptidylprolyl isomerase B (Hs-PPIB; 320861, ACD) and bacterial dihydrodipicolinate reductase (DapB; 320871, ACD) were utilized as positive and negative controls, respectively. The probes were labeled with fluorophore Opal 570 (FP1488001KT, Akoya Biosciences). Subsequently, FFPE sections were stained with anti-langerin antibodies (clone D9H7R; 13650, CST), which were then labeled with Opal 690 (FP1497001KT, Akoya Biosciences) or Opal 520 (FP1487001KT, Akoya Biosciences). Finally, tissue sections were counterstained with DAPI (323108, ACD) and mounted with ProLong Gold Antifade Mountant (P36930, Invitrogen). Images were captured on a TCS SP8 Confocal Microscope (Leica Microsystems) or a LSM900 Confocal Microscope (Carl Zeiss), and they were processed using ImageJ software (NIH). All slides were scrutinized for IL-23p19 or IL-23p40 mRNA signals in morphologically intact LCs and scored independently by 2 researchers in a blinded manner. IL-23p19 and IL-23p40 mRNA signals were identified as red, punctate dots, and expression level was scored as follows: 0 = no staining or less than 1 dot per 10 cells, 1 = 1–3 dots per cell, 2 = 4–9 dots per cell (few or no dot clusters), 3 = 10–15 dots per cell (less than 10% in dot clusters), and 4 = greater than 15 dots per cell (more than 10% in dot clusters). A cumulative score of each tissue sample was calculated as the sum of the individual products of the expression level (scores 0–4) and percentage of cells (scores 0–100) as follows: (A% × 0) + (B% × 1) + (C% × 2) + (D% × 3) + (E% × 4), which ranged between 0 and 400.

### Epidermal LC separation.

To eliminate nonviable cells, epidermal single-cell suspensions were incubated with dead cell removal microbeads (130090101, Miltenyi Biotec) and negatively separated using a LS column (130042401, Miltenyi Biotec). To enrich LCs, viable cells were labeled with epidermal LC microbeads (130095408, Miltenyi Biotec) and positively separated using a large cell separation column (130-042202, Miltenyi Biotec). LC-enriched cell suspensions were stained with anti–mouse MHC-II Ab (clone M5/114; 562363, BD Biosciences) and anti–mouse CD45.2 Ab (clone 104; 109832, BioLegend). LCs (MHC-II^+^CD45.2^+^) were sorted using a Beckman Moflo XDP cell sorter, and the purity was confirmed to be > 95% by flow cytometry.

### Lipid profiling by LC-MS.

Lipids were extracted from isolated LCs using chloroform/methanol (2/1, v/v), dried under nitrogen at 40°C, and resolved in isopropanol/methanol (1/1, v/v) for component analysis on an Ultimate 3000 LC Orbitrap Elite LC-MS/MS instrument (Thermo Fisher Scientific). LC separation was performed on an ACQUITY UPLC HSS T3 column (2.1 × 100 mm, 1.8 μm; Nihon Waters K.K.) using a mobile phase of acetonitrile/water (60/40, v/v; mobile phase A) and acetonitrile/isopropanol (10/90, v/v; mobile phase B) with a flow rate of 0.3 mL/min at 45°C. Both mobile phases contained 0.1% methanoic acid and 10 mmol/L ammonium acetate. The gradient conditions for lipid separation were as follows: 0/30, 2/43, 2.1/55, 12/65, 18/85, 45/100, and 55/30 (minutes/% solvent B). The mass spectrometry (MS) instrument was operated in both positive and negative ion modes as follows: electron spray ionization (ESI) + heater temperature = 300°C, sheath gas flow rate = 45 arbitrary units (arb), aux gas flow rate = 15 arb, sweep gas flow rate = 1 arb, spray voltage = 3.0 kV, capillary temperature = 350°C, s-lens indicates radiofrequency (rf) level = 30%; ESI-heater temp = 300°C, sheath gas flow rate = 45 arb, aux gas flow rate = 15 arb, sweep gas flow rate = 1 arb, spray voltage = 3.2 kilovolt (kV), capillary temp = 350°C, and s-lens rf level = 60%. The obtained data were extracted using LipidSearch software (Thermo Fisher Scientific), normalized based on the number of cells, and arranged into 2-dimensional data matrices including LipidIon, class, FA chains, CalcMz, IonFormula, RT and peak area by Microsoft Excel.

### Low-input mRNA sequencing analysis.

The Smart-Seq protocol was performed on sorted LCs as described previously (19) with some modifications. Briefly, double-stranded cDNA was generated from 2 × 10^3^ to 2 × 10^4^ LCs using a Smart-Seq V4 Ultra Low Input RNA Kit (634894, Clontech) following the instruction manual. The cDNA was amplified over 15 PCR cycles. The cDNA library was generated with a TruSeq DNA Library Prep Kit (fc1212001, Illumina). Clusters were produced with a TruSeq PE Cluster Kit (PE-401-3001, Illumina) according to the manufacturer’s protocol. High-throughput RNA-Seq was performed using an HiSeq 3000 instrument (Illumina). STAR software was utilized for sequence alignment with the preprocessing sequence and reference genome sequence of mice downloaded from the Ensembl database (Mus_musculus.GRCm38.90 downloaded from http://ftp.ensembl.org/pub/release-90/gtf/mus_musculus/). Transcript assembly of mRNA-Seq data was performed by StringTie software. Expression levels for each gene were normalized using the fragments per kilobase of transcript per million fragments mapped (FPKM) method. Clustering of each sample was performed by principal component analysis (PCA) and calculating the Pearson correlation. DESeq2 was applied to identify and analyze DEGs. The cutoffs for DEGs were *P* ≤ 0.05 and fold-change (FC) ≥ 2. Functional annotation of DEGs was conducted using Gene Ontology (GO) and KEGG pathway analyses.

### Data availability statement.

Data sets related to this article can be found at https://www.ncbi.nlm.nih.gov/geo/query/acc.cgi?acc=GSE162274, hosted at GEO.

### Statistics.

Data are presented as mean ± SEM. Statistical analyses were performed with GraphPad Prism software using 2-tailed Student’s *t* tests. A *P* value less than 0.05 was considered significant. For multiple comparisons, *P* values were adjusted for multiple testing using the FDR method.

### Study approval.

All the experiments conducted in this study were approved by the Institutional Research Ethics Boards of Shanghai Skin Disease Hospital (2021-05). All the participants provided written informed consent prior to participation in the study. Handling of mice and experimental procedures were in accordance with the requirements of Animal Care and Use Committee of Shanghai Tongji University.

## Author contributions

XZ was responsible for funding provision, experimental design, data collection, data analysis, manuscript composition, and revision. XL was responsible for experimental design, data collection, and data analysis. YW was responsible for data collection and data analysis. YC participated in the conduction of animal experiments. YH performed LC sorting by flow cytometry. CG participated in the immunohistofluorescence experiments. ZY participated in the conduction of in vitro LC culture experiments. PX participated in the conduction of animal experiments. YD collected normal skin tissues and psoriatic lesions. QSM provided extensive discussion and mentoring over the project. JW supervised the design, reviewed data for all the experiments in this project, and critically reviewed the manuscript. JG provided funding, supervised the design, reviewed data for all the experiments in this project, and critically reviewed the manuscript. YS provided funding, supervised the design, reviewed data for all the experiments in this project, and critically reviewed and revised the manuscript. The authorship order among co–first authors are as follows: XZ (first), XL (second), and YW (third), based on their degrees of contributions.

## Supplementary Material

Supplemental data

## Version 1 07/08/2022

Electronic publication
