# Supplementary material for: Abnormal lipid metabolism in epidermal Langerhans cells mediates psoriasis-like dermatitis
Source: JCI Insight. 2022 Jul 8;7(13):e150223. doi: 10.1172/jci.insight.150223 (PMC9310522; doi:10.1172/jci.insight.150223)
Supplement: Supplemental data [file jciinsight-7-150223-s140.pdf]

Supplementary Data

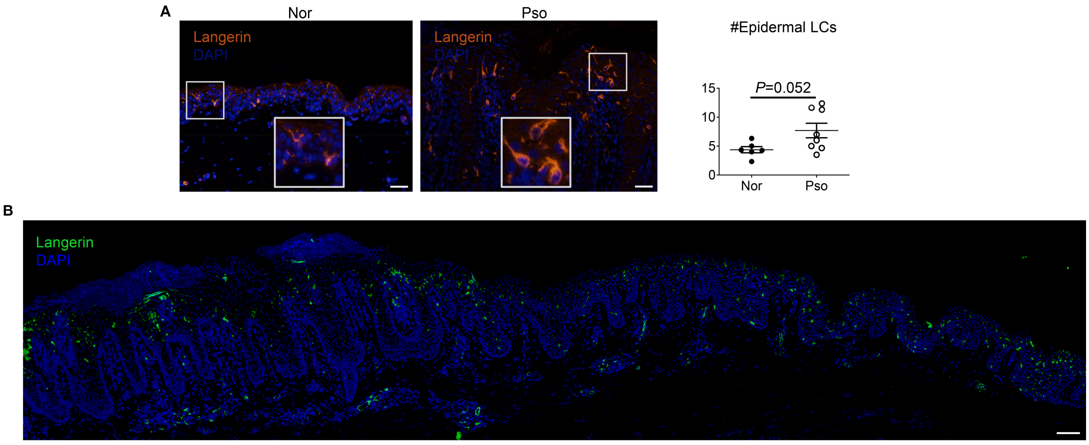

Supplementary Figure 1 Epidermal LCs enlarge in psoriatic lesions

(A) Representative immunofluorescence of LCs (langerin^{+}) and their numbers in FFPE tissue sections of normal human skin and psoriatic lesions (x 200; bar=50μm; n=14, three independent experiments). **(B)** Immunofluorescence of epidermal LC distribution in a FFPE tissue section of the psoriatic lesion and adjacent unaffected skin tissue from one psoriasis patient (x 6; bar=100μm). Two-tailed Student's t-test was performed. The data are presented as mean ± SEM.

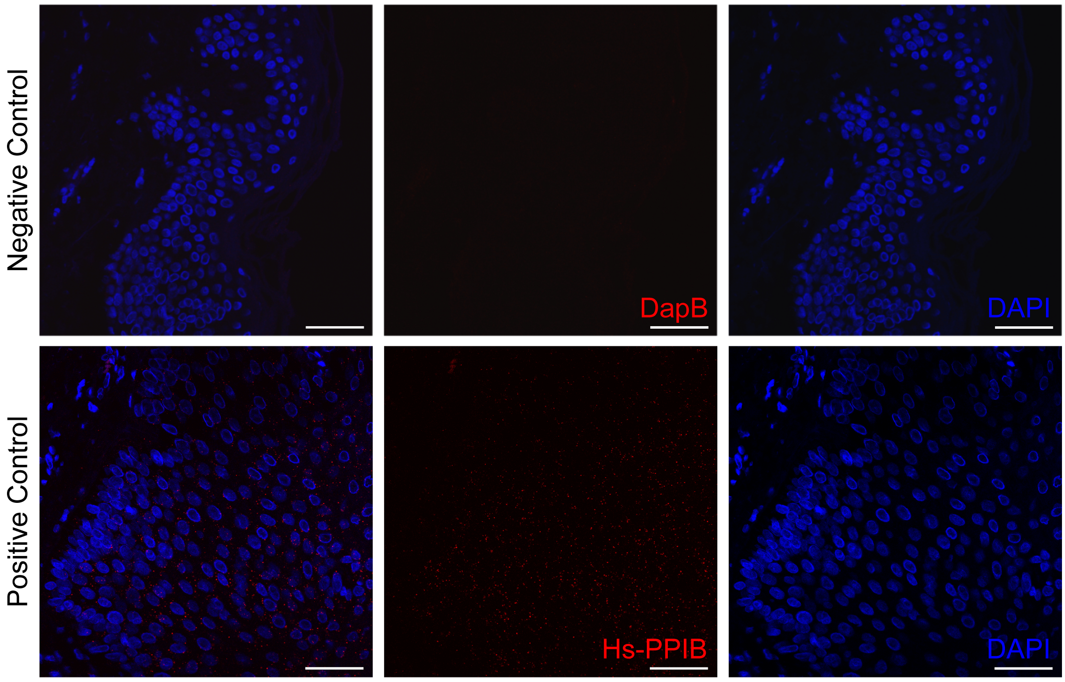

Supplementary Figure 2 RNAscope negative and positive controls

Representative RNAscope to probe DapB (negative control) and Hs-PPIB (positive control) in FFPE tissue sections of normal human skin (x400; bar=50μm).

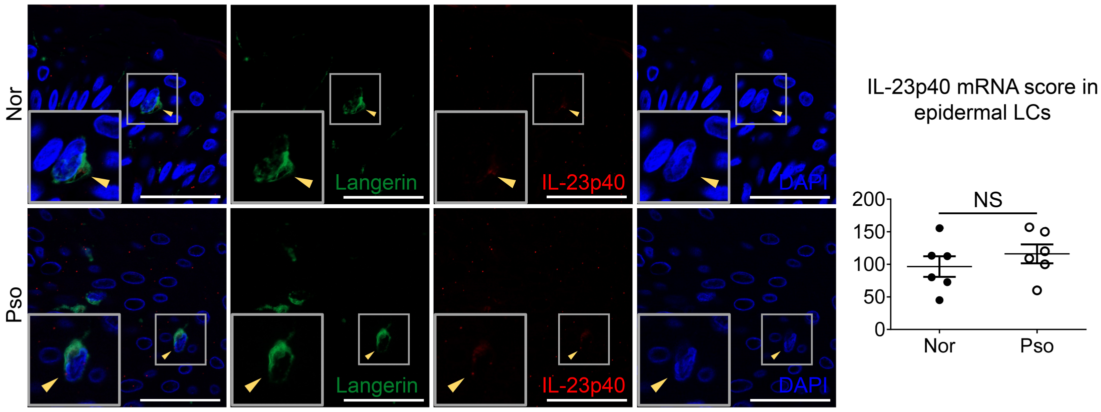

Supplementary Figure 3 The IL-23p40 mRNA level of epidermal LCs from psoriasis patients is equivalent to normal LCs of healthy individuals

RNAscope of IL-23p40 together with immunofluorescence of LCs and IL-23p40 mRNA score in epidermal LCs (x630; bar=40μm; n=12, three independent experiments). Two-tailed Student's t-test was performed. The data are presented as mean ± SEM.

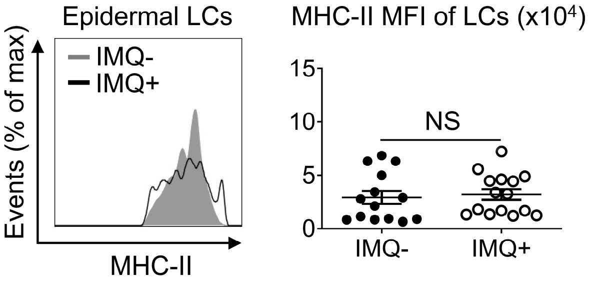

Supplementary Figure 4 Imiquimod-induced psoriasis-like dermatitis did not influence LC expression of MHC-II

Mice were treated as in **Figure 2A**, and epidermal LCs were harvested and stained as in **Figure 2, C-F**. Representative histogram and MFI of MHC-II in epidermal LCs (dark grey filled: IMQ-untreated; black line: IMQ-treated; n=29, seven independent experiments). Two-tailed Student's t-test was performed. The data are presented as mean ± SEM.

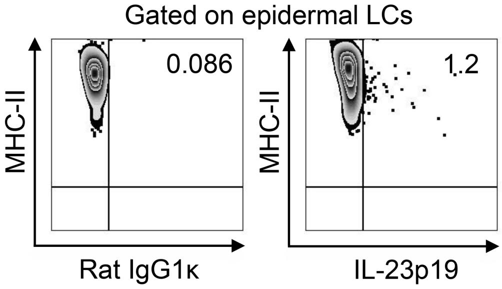

Supplementary Figure 5 Langerhans cells secrete interleukin-23

Epidermal cells freshly-isolated from IMQ- mice trunk skin was cultured in vitro in the presence of Golgi Stop for 4 hours, which were stained with anti-MHC-II, Langerin and IL-23p19 Ab or its isotype control Rat IgG1κ and analyzed by flow cytometry. Representative FACS analysis of Rat IgG1κ^{+} (left panel) and IL-23p19^{+} (right panel) LCs.

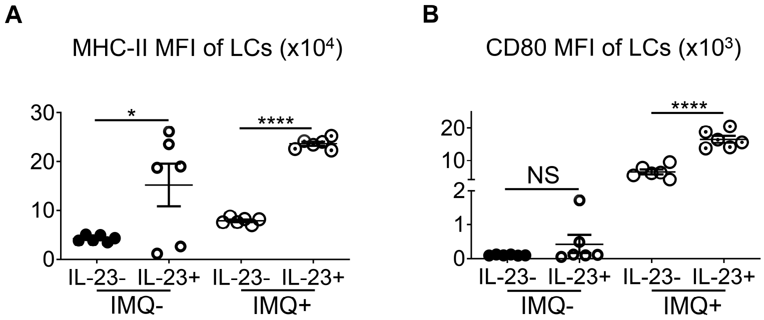

Supplementary Figure 6 Interleukin-23-secreting Langerhans cells are more mature than interleukin-23-non-secreting Langerhans cells

Epidermal cells freshly-isolated from mouse trunk skin of IMQ- and IMQ+ mice were cultured in vitro in the presence of Golgi Stop for 4 hours, which were stained with anti-MHC-II, CD45.2, CD80 and IL-23p19 Ab, and analyzed by flow cytometry. MHC-II **(A)** and CD80 **(B)** MFI of epidermal LCs from IMQ- and IMQ+ mice (n=12, three independent experiments). Two-tailed Student's t-test was performed. Tests were considered significant with $P<0.05$ after multiple testing adjustments by the FDR method. The data are presented as mean ± SEM. *$P<0.05$, ****$P<0.0001$.

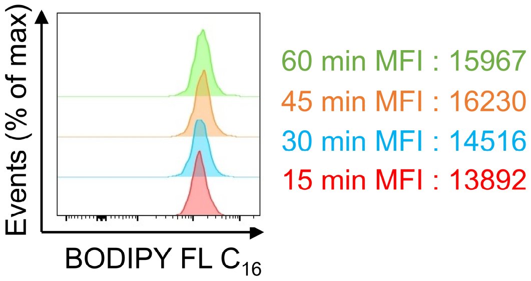

Supplementary Figure 7 Langerhans cells achieve maximum uptake of BODIPY FL C_{16} between 30 to 45 minutes

Epidermal cells freshly-isolated from mouse trunk skin were incubated with BODIPY FL C_{16} (1μM) at 37 ℃ for 15 minutes (red), 30 minutes (blue), 45 minutes (orange) and 60 minutes (green), which were stained with anti-MHC-II and anti-CD45.2 Ab and analyzed by flow cytometry.

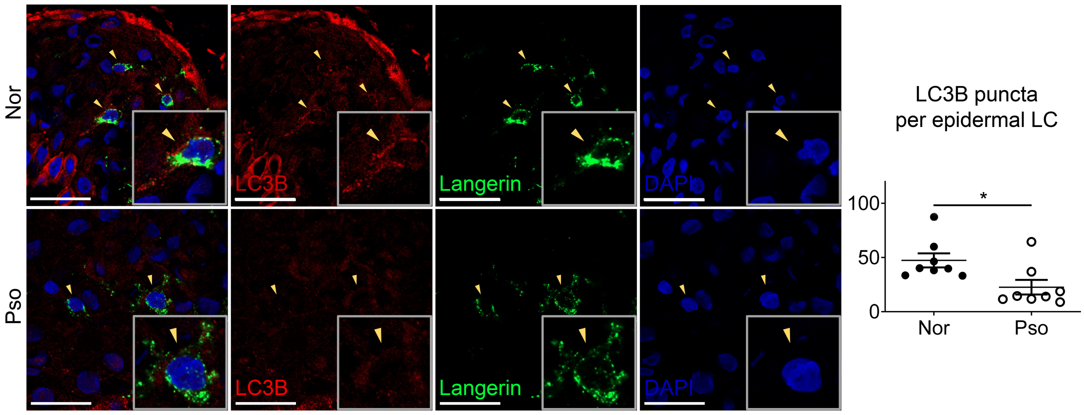

Supplementary Figure 8 Decreased LC3B expression in epidermal LCs of psoriasis patients

Representative immunofluorescence of LC3B expression and the quantities of LC3B puncta in LCs from frozen sections of normal human skins and psoriatic lesions (x630; bar=30μm; n=16, three independent experiments). Two-tailed Student's t-test was performed. The data are presented as mean ± SEM. *P<0.05.

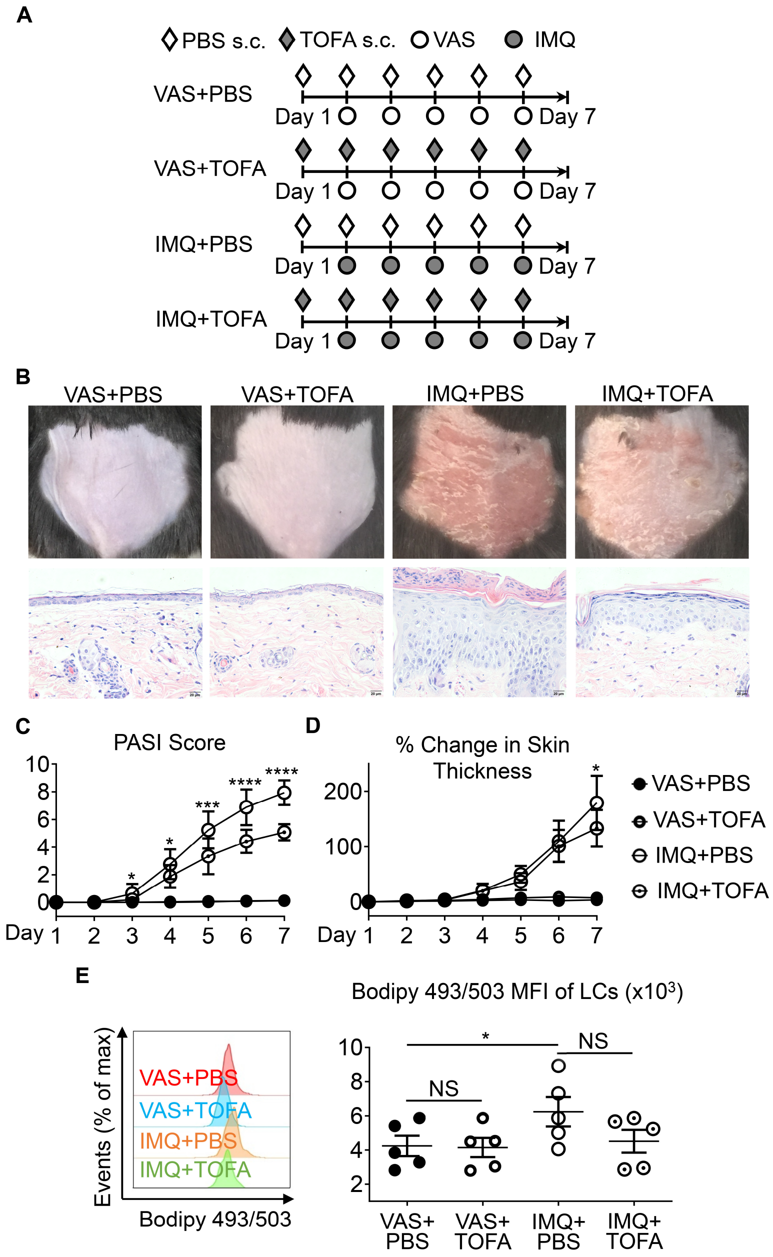

Supplementary Figure 9 Subcutaneous administration of TOFA alleviated IMQ-induced psoriasis-like skin inflammation

Mice received a daily topical dose of 62.5 mg of 5% imiquimod (IMQ) cream or Vaseline (VAS) on shaved back for 5 consecutive days. 5-tetradecyloxy-2-furoic acid (TOFA; 5mg/kg body weight) or phosphate buffer saline (PBS) was administered subcutaneously one day before the application of VAS or IMQ cream, which continued for 6 successive days. **(A)** Schematic representation of experimental procedures. **(B)** Representative photographs of skin lesions (upper panel) and their H&E staining (lower panel; x400; bar=20μm). **(C)** Evaluation of skin inflammation severity using PASI score (n=68, five independent experiments). **(D)** The percentages of skin thickness increase measured by vernier calipers (n=68, five independent experiments). **(E)** Histogram and MFI of Bodipy 493/503 in epidermal LCs (n=20, three independent experiments). For Supplementary Figure 9C, D, one-way ANOVA was utilized to analyze the differences between four groups, and two-tailed Students t-test of the difference between IMQ+PBS group and IMQ+TOFA group was performed after multiple testing adjustments by the FDR

method, which statistical significances were annotated. For Supplementary Figure 9E, two-tailed Student's t-test was performed, and tests were considered significant with $P<0.05$ after multiple testing adjustments by the FDR method. The data are presented as mean ± SEM. *$P<0.05$, ***$P<0.001$, ****$P<0.0001$.

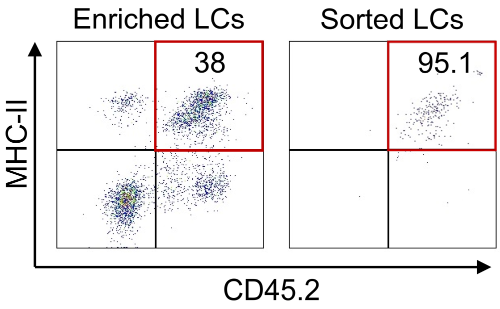

Supplementary Figure 10 Langerhans cell purity after sorting

Freshly-isolated epidermal cells underwent dead-cell removal and LC enrichment using microbeads, and stained with anti-MHC-II and anti-CD45.2 Ab. Epidermal LCs (MHC-II^{+} CD45.2^{+}) were sorted from LC-enriched epidermal cell suspensions by flow cytometry. Representative FACS analysis of LC-enriched epidermal cell suspensions and isolated LCs.

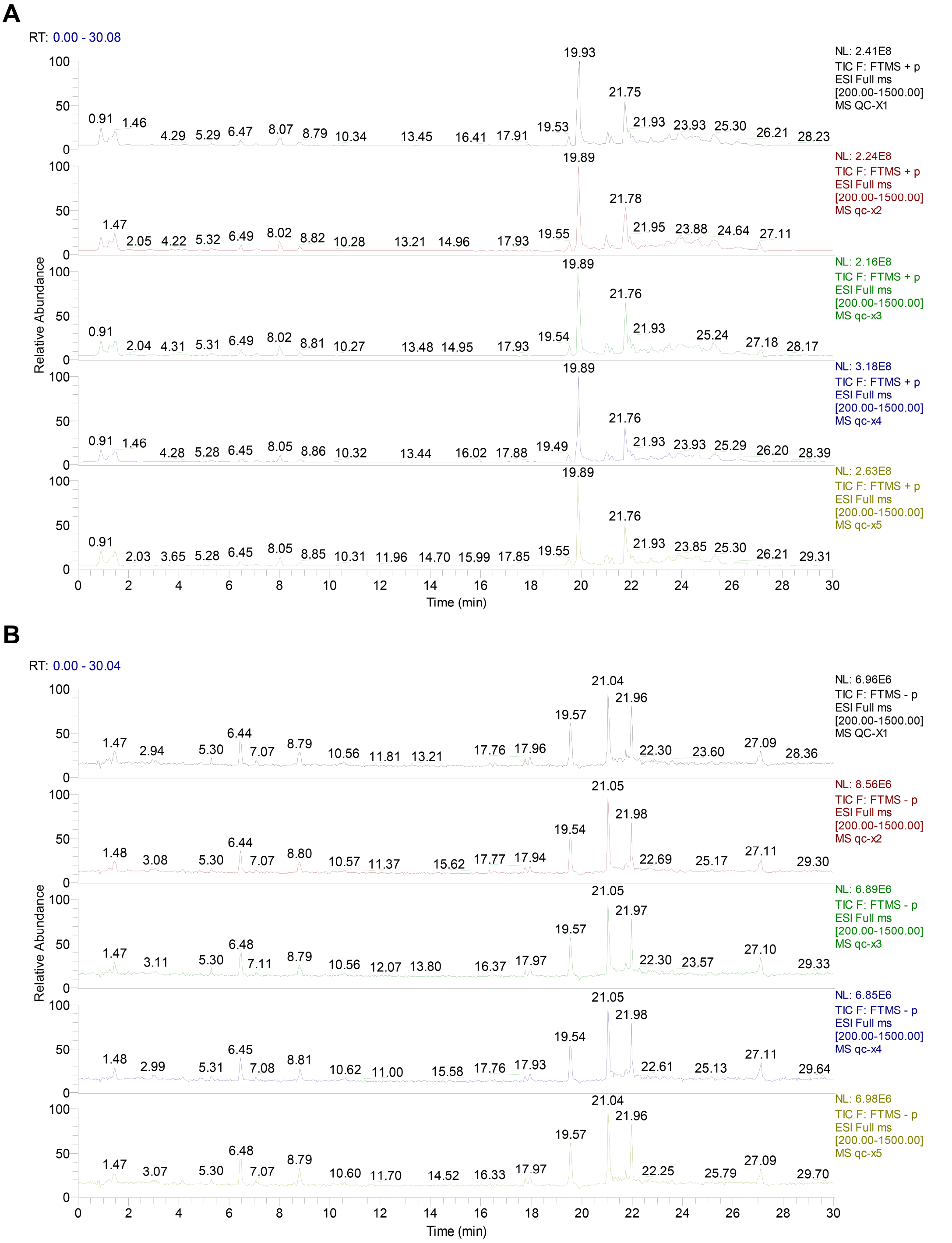

Supplementary Figure 11 Total ion chromatograms of quality control samples

The total ion chromatogram of quality control samples under the positive ion (ESI+) mode **(A)** and negative ion (ESI-) mode **(B)**.

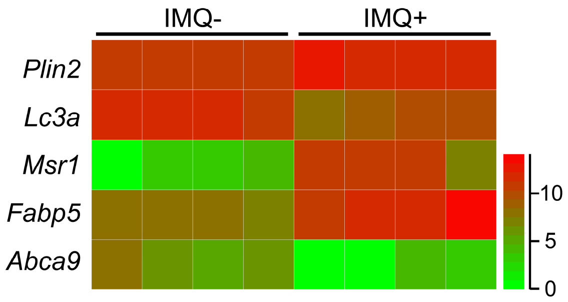

Supplementary Figure 12 Heatmap of DEGs in epidermal Langerhans cells

Epidermal LCs were freshly-sorted from IMQ- and IMQ+ mice and underwent low-input mRNA sequencing analysis as in **Figure 6**. Heatmap of *Plin2*, *Lc3a*, *Msr1*, *Fabp5*, and *Abca9* gene expressions in LCs.

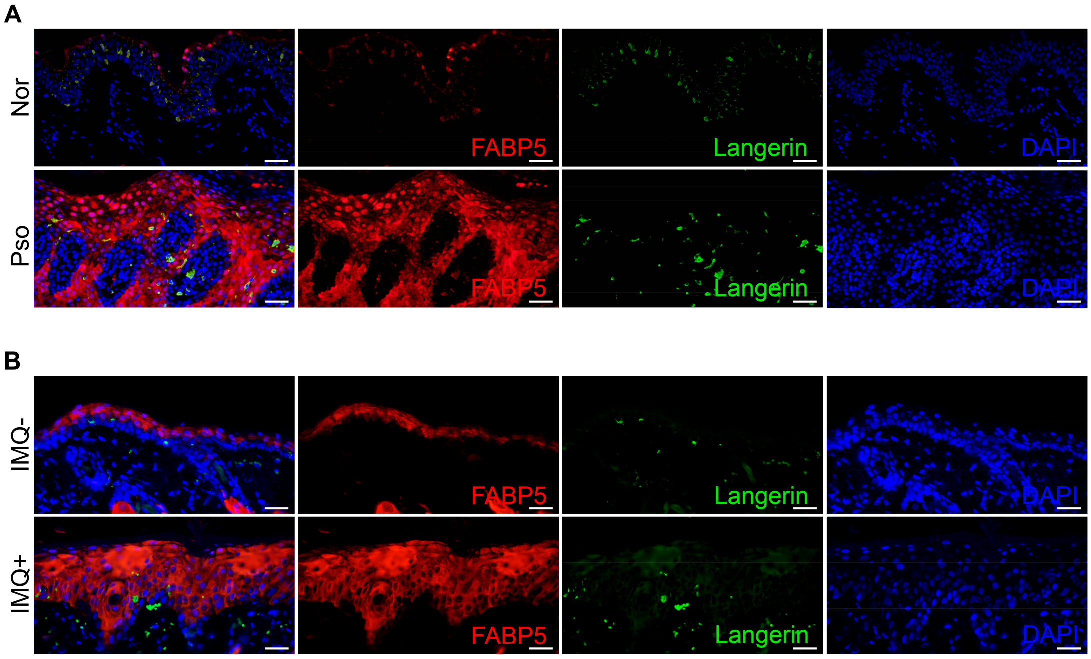

Supplementary Figure 13 Epidermal LCs of psoriatic lesions and imiquimod-induced psoriasis-like skin have a higher level of FABP5 expression

(A) Representative immunofluorescence of FABP5 expression in LCs from FFPE tissue sections of normal human skin and psoriatic lesions (x400; bar=50μm; n=10, two independent experiments). **(B)** Representative immunofluorescence of FABP5 expression in LCs from back skin tissue of IMQ- and IMQ+ mice (x400; bar=50μm; n=12, two independent experiments).

Supplementary Table 1 The summary of raw RNA sequencing data

It showed the summary of RNA sequencing data of 8 samples, including raw reads, clean reads, and percentage of clean reads as well as Q20% and Q30% (the percentage of bases with Phred values > 20 and > 30)

Sample	Raw Reads	Clean Reads	Clean Reads%	Q20%	Q30%
LC WT1	62294592	56980606	91.47%	95.01%	90.67%
LC WT2	74742522	72550786	97.07%	96.30%	92.61%
LC WT3	80486898	78262056	97.24%	94.56%	88.86%
LC WT4	88174704	85223750	96.65%	95.06%	89.66%
LC IMQ1	77731196	74677134	96.07%	94.65%	88.50%
LC IMQ2	69286224	65610554	94.69%	94.50%	88.45%
LC IMQ3	56106510	50923288	90.76%	93.15%	86.76%
LC IMQ4	55336008	53485118	96.66%	95.75%	90.95%

Supplementary Table 2 Gene ontology annotation enriched in the epidermal Langerhans cells from imiquimod-induced psoriasis-like skin in comparison with the Langerhans cells from normal skin by gene set enrichment analysis

Name	ES	NES	Nom p-val	FDR q-val
GO_STRUCTURAL_CONSTITUENT_OF_RIBOSOME	0.50	2.50	<0.001	0.000
GO_CCR_CHEMOKINE_RECEPTOR_BINDING	0.63	2.31	<0.001	0.005
GO_G_PROTEIN_COUPLED_CHEMOATTRACTANT_RECEPTOR_ACTIVITY	0.66	2.28	<0.001	0.004
GO_NUCLEOTIDE_RECEPTOR_ACTIVITY	0.65	2.18	<0.001	0.007
GO_CHEMOATTRACTANT_ACTIVITY	0.56	2.12	<0.001	0.012
GO_NADH_DEHYDROGENASE_ACTIVITY	0.51	2.09	<0.001	0.015
GO_CHEMOKINE_RECEPTOR_BINDING	0.52	2.04	<0.001	0.021
GO_LOW_DENSITY_LIPOPROTEIN_PARTICLE_BINDING	0.62	1.97	<0.001	0.031
GO_CYTOKINE_ACTIVITY	0.37	1.96	<0.001	0.029
GO_CHEMOKINE_BINDING	0.54	1.93	<0.001	0.034
GO_C_C_CHEMOKINE_BINDING	0.54	1.91	0.006	0.036
GO_PROTEIN_LIPID_COMPLEX_BINDING	0.52	1.86	0.006	0.047
GO_PEPTIDE_RECEPTOR_ACTIVITY	0.39	1.84	<0.001	0.047
GO_CHEMOKINE_ACTIVITY	0.48	1.83	0.006	0.045
GO_CYTOKINE_RECEPTOR_ACTIVITY	0.38	1.83	<0.001	0.044
GO_OLFACTORY_RECEPTOR_ACTIVITY	0.39	1.79	<0.001	0.052
GO_PROTEASE_BINDING	0.37	1.78	<0.001	0.054
GO_PATTERN_RECOGNITION_RECEPTOR_ACTIVITY	0.51	1.75	0.006	0.060
GO_LIPOPOLYSACCHARIDE_BINDING	0.50	1.72	0.006	0.068
GO_CYTOKINE_RECEPTOR_BINDING	0.33	1.71	<0.001	0.067
